# The Lacanian Concept of Cut in Light of Lacan's Interactions with Maud Mannoni

**DOI:** 10.3389/fpsyg.2017.02177

**Published:** 2017-12-14

**Authors:** Laure Razon, Olivier Putois, Alain Vanier

**Affiliations:** ^1^Université de Strasbourg, SuLiSoM EA 3071, Strasbourg, France; ^2^Department of Psychiatry, Mental Health and Addictology, Strasbourg University Hospital, Strasbourg, France; ^3^CRPMS EA 3522, Department of Psychoanalytic Studies, Paris Diderot University, Sorbonne Paris Cité, Paris, France

**Keywords:** cut, psychosis, family, Lacan, Maud Mannoni, mental retardation, maternal fantasy, object *a*

## Abstract

This article sets out to shed light on the Lacanian concept of cut (introduced in 1961–1962): it refers to the symbolic (i.e., linguistic) operation which produces the object *a* and thereby enables separation, through which the subject emerges. To that effect, we show how this concept benefitted from Lacan's interactions with Maud Mannoni (1923-1998), who focused on clinical situations where implementing a cut in the subject's environment is the only way to enable a separation between the child and the Other. Lacan first drew on Mannoni's clinical elaborations about the retarded child's alienation to the maternal fantasy: when the mother's unconscious doesn't leave room for the cut, it prevents the separation through which the child could become a subject. Lacan generalized this in the late 1960s: he broadened Mannoni's alienation to the maternal fantasy to characterize a type of child symptom, where children become their mother's non-separated, de-phallicized object *a*. Then, in the 1970s, Mannoni proposed an original theoretico-clinical setting to address the configurations where the object *a* isn't separated: in the splintered institution, the team follows on projects of activities (professional, personal, etc.) outside the institution voiced by children who haven't previously encountered the symbolic cut, by helping them realize these external projects. By thus acknowledging their attempts at establishing a cut and giving them consistency, the splintered institution helps them psychically elaborate separation.

## Introduction

Lacan introduced the notion of cut in the early 1960s: its refers to the operation (broader than castration, both in scope and in nature) through which the infant's mothering figure (the maternal or primordial Other) enables psychical separation, by introducing the infant to a representation of him as symbolically distinct from her. Receiving this representation enables the infant to ultimately acknowledge his separation from her body; in Lacan's view, only then does the infant become a subject.

In this paper, we shed light on the theoretical and clinical fruitfulness of this concept of cut by addressing it from a specific perspective: that of the cases where the paradigm representation enabling the cut—the Name-of-the-Father, Lacan's rewriting of the Oedipus complex—isn't transmitted to the infant, thereby precluding the cut to be established in the child's psyche. How should one characterize such cases? Our hypothesis is that Maud Mannoni's clinical and theoretical elaborations on the psychotic dimension of such cases, which took place in a constant exchange with Lacan, provided him with crucial clinical material to conceptualize such situations and give the concept of cut its final shape. In return, she used Lacan's concept of cut in a very original fashion, by putting together a new type of institution trying to enable the emergence of the cut within the unconscious of children not heretofore presented with the Name-of-the-Father, by providing them with representations to implement the cut according to their structure (neurotic, psychotic, perverse, or autistic).

We first lay out the concept of cut as Lacan established it in the early 1960s, and insist on its paradigm condition—the Name-of-the-Father. We then show how Lacan first drew on Mannoni's clinical elaborations about the retarded child's alienation to the maternal fantasy: when this fantasy doesn't leave room for a paternal cut, it prevents the separation through which the child could become a subject. He expanded on this in the late 1960s, in the first “Note on the Child:” the child can come to embody the object of the maternal fantasy and thereby become the mother's non-separated, de-phallicized object *a*—which these cases help characterize. Finally, we show how Mannoni, in the 1970s, proposed an original theoretico-clinical setting to address the configurations where the cut hasn't been implemented due to a lack of paternal metaphor, and the object *a* therefore hasn't been separated. The splintered institution is a therapeutic setting which assumes the cut by taking up desires of outside projects expressed by its young hosts: children are accompanied outside to participate in projects wherein they identify with a different representation of themselves, and thereby experience themselves as separated.

## The cut according to Lacan, in the early 1960s

### Alienation, a precondition to the cut

The Lacanian concept of cut, or better the “function of the cut” (Lacan, 1998a, p. 206), refers to a structural sequence: it is not an event taking place at a determinate time in individual development, but a logical moment in the constitution of the subject. It is the result of the encounter between a real infant and the family and social configuration with which the infant is presented by the Other. (The term Other refer to the infant's primitive environment, the mother or the mothering adult).

The function of the cut is to enable the constitution of the subject through separation with the Other—we will come back to this point in detail. This separation has a prerequisite: the first sequence of the constitution of subjectivity, which Lacan calls “alienation” (Lacan, 1998a). Upon meeting the Other, the infant is exposed to language: in responding to his cries and behavior, the Other names him, he refers to him. In this exchange, the infant comes to realize that in order to become a subject, he must use what Lacan calls signifiers (characteristic of the symbolic order)—they are the discrete elements of language considered as different sounds, independently of their usual socially determined meaning. In particular, the infant gradually sees that in order to interact with the Other, he must endorse the signifier through which the Other designates him, called the primary or master signifier (S_1_)—the future object of primary repression. Lacan calls this operation ≪ alienation ≫ not only because the infant, generally speaking, receives the discourse of the Other through his speech, but most importantly because in this encounter with the Other, he receives a specific signifier S_1_, the assumption of which conditions his existence for the Other, and in particular communication with him. The signifier “functions as a signifier only by reducing the subject in question to being no more than a signifier, to petrify him in the same movement it calls the subject to function, to speak, as subject” (Lacan, 1998a, p. 207, mod. tr.).

The question then becomes, in order for the infant to become a subject properly speaking, to know what he represents for the Other, what the Other ≪ wants from him ≫ (Lacan, 2014)—that is, what is the meaning of this S_1_ which designates him? Since a single signifier, as such, has no meaning, S_1_ can only acquire one by becoming part of a series of other signifiers coming from the Other (Lacan calls S_2_ the ensemble of these additional signifiers). In other words, alienation does not suffice to constitute the subject because, far from enabling the infant to gain access to a knowledge of what he is, it instead opens him to a never-ending series of equivalences between S_1_ and S_2_. It is this series of equivalences that enables the explorations of infantile curiosity (Lacan, 1998a, part 3), and more generally accounts for the effects of metaphor and metonymy through which formations of the unconscious can be interpreted.

### The cut and the desire of the other

It is at this very point, in these “intervals which cuts between the signifiers” S_1_ and S_2_ (Lacan, 1998a, p. 214, mod. tr.) that the second part of the structuring effect of the signifier on the subject comes to play: that is, the function of the cut. Endorsing S_1_ opens a ≪ *Spaltung* [splitting] in the subject ≫ (Lacan, 1998a, p. 63): he becomes cut, ≪ divided ≫ because the relationship between S_1_ and S_2_, far from being immediately obvious (since they both lack intrinsic meaning), only makes sense through metaphorical and metonymical equivalences within a specific network of signifiers which characterizes the constellation laid out by the Other specific to every subject. The unconscious effect of any S_1_ will thus depend, for each subject, on the S_2_ with which it is paired.

Thus, the constitution of the subject requires the cut in addition to alienation because the interval between S_1_ and S_2_, the repressed primary signifier and its equivalents in the discourse of the Other, draws on equivalences that are specific to the discourse of every particular Other, the desire of whom is the key to understand these equivalences. “It is in the interval between these two signifiers that resides the desire offered to the mapping of the subject of the discourse of the Other, of the first Other he has to deal with, let us say, by way of illustration, the mother” (Lacan, 1998a, p. 218). Thus the cut enables the constitution of the subject by leading the infant to wonder what the desire of the maternal Other is, what she wants from him (this question drives the analytic process, which will regressively lead to discover what one's S_1_ is, as well as the ultimately arbitrary, contingent nature of its equivalences with S_2_). “It is in so far as [the subject's] desire is beyond or falls short of what she says, of what she hints at, of what she brings out as meaning, it is in so far as his desire is unknown, it is in this point of lack, that the desire of the subject is constituted” (Lacan, 1998a, p. 218).

### The fort-da: separation as an effect of the cut, and the introduction of object *a*

Lacan believed that the subject's question about the desire of the Other, located in the cut between signifiers, can be paradigmatically illustrated by the sequence of the Freudian reel play (the ≪ *fort-da* ≫). This sequence illustrates the dependence of the subject's being on the desire of the Other.

In *Beyond the Pleasure Principle* (Freud, 2001, written in 1920), Freud describes how an 18-months infant—most likely his grandson Ernst—symbolizes the absences of his mother by playing with a reel attached to a string, thereby gradually enabling the introjection of the absented parent. Upon throwing the reel away from his cradle, he gives vent to a loud ≪ o-o-o-o ≫ (which means “*fort*” [away] in German), before pulling it back into view and hailing its appearance with a gleeful “Da !” (“There !”). In analyzing the infant's play, and in particular the substitution of the reel to the absent mother, Freud insists on the psychical achievement of becoming able to acknowledge her absence and to withstand it, by figuratively becoming the agent of this absence (in throwing the reel away).

But for Lacan, compensating for her absence by converting passivity into activity is “of secondary importance” (Lacan, 1998a, p. 62). He starts by pointing that the second step of the play (the “da!”), by introducing a phonemic alternation, represents the comings and goings through a pair of signifiers, S_1_ (“o-o-o”) and S_2_ (“da!”). Thus, the *fort-da* play presents us with the paradigmatic example of the effect of the cut (S_1_-S_2_ pair) on the subject—that is, separation. The physical disappearance of the maternal Other (endowed with a breast) can only become meaningful for the subject insofar as, by drawing on the “o-o-o/da!” pair, he is led to wonder what type of object he is in the eye of this Other who keeps coming and going. In other terms, it is the phonemic opposition that enables the infant to wonder what takes place inbetween S_1_ and S_2_–which refer to absence and presence. Therefore, in throwing the reel, the infant stages this very question: the reel represents him as an object separated from the body of the Other—Lacan calls it “object *a*,” a non-empirical object which causes the desire of the Other once it is separated from his body (Lacan, 2014). By thus staging the Other separating from the object (reel-baby) and then coming back to it, the infant understands the comings and goings of the Other as manifestations of her desire, on which the infant draws in order to and correspondingly become a desiring subject. For in this sequence, he realizes that he lacks what the desire of the Other can give him, namely the bodily part (especially the breast) experienced as separated from him, which in turn becomes a separated object *a* for him (Lacan, 1998a, p. 62). Thus the *fort-da* play, which draws on the function of the cut in its simplest expression (“o-o-o”/ “da!”), produces a separation by leading the infant to wonder what object *a* he is to the Other and thereby to develop a desire echoing that of the Other (*ibid*.).

The question then becomes to know what conditions, on the side of the Other, the subject's access to the function of the cut, and the consecutive representation of a separation with the Other and the corresponding loss of the object *a*. To address this question, we start by examining the paradigmatic condition to access the cut (the paternal metaphor), and the corresponding version of the cut: castration.

### The paradigmatic condition of the cut on the side of the other: the paternal metaphor, and its absence

Since the function of the cut puts to work a relation of metaphorical and metonymical substitution between S_1_ and S_2_, the paradigmatic condition of its implementation in the infant's psyche is what Lacan calls the paternal metaphor (Lacan, 1966, 1998b,c). This concept, which refers to Lacan's rewriting of the Oedipus complex, helps specify an Oedipal mode of representing oneself as separated from the Other and thereby as object *a* of their desire.

The paternal metaphor is what enables the child to understand the mother's (or mothering figure's) absence as an Oedipal separation: this metaphor refers to the effect of the introduction, by the Other, of a specific signifier called the Name-of-the-Father (Lacan, 1966, p. 557). This signifier is meant to introduce the infant to the symbolic operation of castration, which entails two things. First, the Other's desire also has another object, referred to by this signifier (it can be the real father, but anyone can be in this position); therefore, the infant doesn't play the role of a phallus, that is of an object fully satisfying the mother's desire. And second, the Other's desire is nonetheless related to the infant, since its object stands in a symbolic relation of filiation with him. The presence of a Name-of-the-Father on the side of the Maternal Other thus conditions the infant's access to the Oedipus, and it plays a role even before the infant's birth by largely contributing to lay out the coordinates of his future subjective structuration. For if the Name-of-the-Father is no guarantee that the infant will be neurotic, it at least presents him with a triangular configuration which can protect him from depending exclusively on the desire of an almighty mother (with its potential arbitrariness), thanks to the presence of another object of desire on her part: her disappearance in the *fort-da* can be understood by the infant as a manifestation of her desire for someone else.

Thus, insofar as the Name-of-the-Father indicates both that the mother's desire has another object and that this object is nonetheless related to the infant, the presence of this signifier in the Maternal Other enables the child's primary identification to the father: by making it possible for the infant to acknowledge the presence of another object for the Mother's desire, this signifier enables him to becomes a representative of the Name-of-the-Father (this is the positive side of symbolic castration for the infant: while he is no longer the mother's phallus, he becomes in exchange a paternal representative). Thereby, the Name-of-the-Father crucially determines the content of the infant's S_1_, and will thus help him investigate his origin in the Other, i.e., his S_2_ (constitution of infantile sexual theories, and of the primal scene fantasy). It thus becomes manifest that the function of the cut will paradigmatically rely on the paternal metaphor, and that castration is therefore to be understood as the Oedipal version of the cut: this metaphor will enable the subject to question the Other's desire with respect to him by establishing and binding the gap between S_1_ and S_2_ through metaphorical and metonymical equivalences, thereby grounding the discourse concerning the origin of the infant in the desire of a parental couple (This does not entail that the infant would access the Oedipal conflicts at the time of the *fort-da* or other absence/presence-staging plays—roughly around 18 months—, but that the infant's experience of the *fort-da* as an introduction to an Oedipal structure will largely depend on the Oedipal structuration of the Other's psyche).

Hence when the Maternal Other's psyche presents the infant with the paternal metaphor, the effect of the cut (the separation of infant as object *a* from the mother) is, so to speak, compensated for by the emergence of another bond between infant and Other: by being acknowledged by the latter as a result of the encounter between her desire and the Name-of-the-Father, the infant becomes phallicized. That is, he is desirable as a representative of her encounter with the Name-of-the-Father, and not only as an object of her own desire. From this moment on, separation also means conjunction: the infant hereafter exists as a subject embedded in a symbolic lineage, and no longer as the sole object of the mother's desire—this phallicization is thus the symbolic gain of the Oedipal version of the cut, to which castration refers.

Correspondingly, the Other's *lack* of acknowledgment of the Name-of-the-Father (and the consecutive lack of paternal metaphor in the discourse transmitted to the infant) will lead the mother to make him her sole object of desire. He becomes a non-phallicized object *a*, which does not depend on the desire of a couple—be it the psychical couple of the mother's Oedipus complex. In this configuration, the infant does not receive the Oedipal discourse-mediated representations which could enable him to access the function of the cut, and thereby separate himself psychically from the mother: her absence isn't understood against the background of an Oedipal triangulation, but as a definitive loss, experienced by the infant as a fragmentation of his own body (characteristic of psychotic anxiety). This type of psychical organization is characteristic of psychosis: the subject as object *a* doesn't experience himself as separated from the Other's body. Lacan does not mean that a maternal Other who does not present his infant with a the Name-of-the-Father is psychotic, since many clinical configurations can prevent her from presenting a Name-of-the-Father to the infant (depressive breakdown, temporary depersonalization, effects of early mother-infant interactions in the context of neuronal or bodily conditions, etc.). But the subjective coordinates in which the infant is thus introduced are psychotic-like, since the S_1_ through which the Other refers to him isn't paired with an S_2_ representing an Oedipal discourse about his origin in the desire of a couple, giving him a specific, distinct position by separating him from an almighty maternal Other.

How can an infant be introduced to the function of the cut when he hasn't initially been presented with the paradigmatic symbolic condition of the cut—namely, the mother-transmitted paternal metaphor? Before examining how Maud Mannoni provided a theoretically and clinically original response to this question (Part 3), we will examine some of the subjective effects of the lack of an Oedipal S_2_, in order to better understand both (1) what a subject not heretofore exposed to the paternal metaphor needs to access the function of the cut, and (2) what such a cut would amount to.

The lack of Oedipal S_2_ can refer to a variety of specific positions attributed to the subject as object *a* by the Other. The first part of Maud Mannoni's seminal contribution lies, in the 1950s and 1960s, in the clinical exploration of one of these subjective positions: namely, the one underlying what was then called mental retardation, or deficiency (we will use this terminology in spite of its connotations, as it was of common use at that time). Lacan, who was her analyst and encouraged her to publish her first book on the subject, underlined as early as in the *Seminar 11* that the great merit of her work was to shed light on the “psychotic dimension” (Lacan, 1998a, p. 238) of mental retardation. This dimension comes from the “reduction” of the child to “being no more than the support of her desire in the most obscure term” (Lacan, 1998a, p. 237, mod. tr.): in the absence of paternal metaphor, the retarded infant, child, or adolescent is often caught up, as Mannoni phrased it, in the maternal fantasy. We will now develop this point.

## The 1950s and 1960s: from the retarded child to the maternal fantasy

Before expanding on the role of the maternal fantasy, we will briefly recall the context of Mannoni's encounter with Lacan. He wanted her book to be the first in his newly inaugurated collection “Le Champ Freudien” (*The Freudian Field*) because it was showing, in the clinical domain of mental retardation—heretofore quite neglected by psychoanalysts—the relevance of the Lacanian function of the cut, by focusing on the effects of the lack of an Oedipal cut (i.e., castration) on the constitution of the subject.

### Mannoni's encounter with Lacan: the psychotic dimension of mental retardation

Lacan noticed Mannoni's work in the 1950s, by attending conferences and reading papers mostly focused on psychoses, about which Françoise Dolto (Lacan's other famous child analyst pupil) hadn't done much work—focused as she was on the vicissitudes of Oedipal configurations. He immediately suggested that Mannoni undertake a cure with him; in parallel, an uninterrupted clinical exchange began: “he would give me all of his Seminars and I was ‘transformed’ by what he wrote” (Didier-Weill, 2001, p. 174); “it was my clinical work he was interested in…he would keep asking about it to know more” (Didier-Weill, 2001, p. 172).

In this first period of their interactions, he encouraged her to share her clinical work on the unconscious family stakes of mental retardation and psychosis: “with his support, I wrote in one go *The Retarded child and the Mother: a Psychoanalytic study”* (Mannoni, 1988, p. 42), published in 1964. Lacan chose this book to open his newly established collection, “The Freudian Field” (Le Seuil editors); it would soon be completed by *The First Meeting with the Psychoanalyst* (1965).

Looking back at what she wanted to show, she wrote:

“The child creates the psychotic response along with someone else; the more he will feel his environment approves of the seriousness of his ‘condition’, the more he will try and ‘adjust’ to the character in which he chose to alienate himself. Raised in the middle of an adult discourse focused solely on his case, the ‘ill child’ has no other solution but to disappear as a subject in order to fully become the illness with which he is equated” (Mannoni, 1970, p. 40).

Starting from the clinical hypothesis according to which “the child's unconscious is often to be sought after in the parent's unconscious” (Mannoni, 1981, p. 77), she notices that, in the psychotherapeutic care of so-called retarded children, psychiatric diagnosis can act as a screen with respect to the contribution of the “psychotic dimension” (Lacan, 1998a, p. 238) of the mothering parent's unconscious to the child's retardation. In other words, this diagnosis can be used as a family defense—with the other parent's frequent tacit consent—to ward off the question of whether the desire of the Other is aimed at a paternal figure, and replace it with a dual mother-infant relationship: the unconscious legitimization by the diagnosis prevents the implementation of the function of the cut and its separating effect. In this context, the child is cornered into adopting the subjective coordinates he is thus being offered, by endorsing this S_1_ which reduces him to the status of dependency characteristic of mental retardation. The diagnosis *qua* S_1_ saturates the coordinates of the child's subjectivization possibilities: he remains alienated within a psychotic-like system of coordinates devoid of any symbolic cut which would enable the mother to view him as a separated being, by relating him to the Name-of-the-Father.

“When the symptom [of retardation, in the psychiatric sense of the word] has become the subject's only means of communication, he holds onto it. It is his language, and he wants it to be acknowledged as such” (Mannoni, 1998, p. 94).

This exchange with Lacan sheds light on Mannoni's characterization of her work in the 1950s and 1960s: “retardation and psychosis are alike” (Mannoni, 1981, p. 84). This intuition can be traced back to the clinical sensitivity derived from Mannoni's childhood experiences: her family reacted to the bewilderment and sense of loss she experienced upon leaving her beloved nurse in Sri Lanka (where her father was the consul of Belgium) by calling her “the retard” (cf. Razon, 2012). Her own subsequent personal trajectory, and her encounter with Lacan, would lead her to elaborate a therapeutic perspective to escape alienating family dynamics: helping retarded children to distance themselves from the position in which they are kept by their environment requires to investigate the psychotic dimension of the parental unconscious, characterized by a lack of cut.

But elaborating a therapeutic perspective doesn't mean that she sought to establish a parental or maternal causality (and thereby, responsibility) at the root of mental retardation—be it in her theorizing or in her clinical work. She tried to shed light on the dimensions of the family's psychical dynamics which prevent the child from developing as well as he can, and which can be influenced through appropriate therapeutic work focused on the establishment of a cut. She always refused to view the parents as guilty of their child's condition, as one of us (AV) has witnessed on a constant basis during 15 years of work alongside her.

Correspondingly, she did not neglect the importance of organic causality in accounting for the child's condition: speaking of a young patient, she writes that her retardation “seemed to have an undeniable organic basis” (Mannoni, 1981, p. 50). She thus remained faithful to Freud's legacy: in the Preface of the Three *Essays*, he draws on the notion of complemental series to stress that the biological dimension of the symptom should always be questioned, alongside the psychical. Therefore, Mannoni's view can be seen as belonging to what is nowadays called a multi-factorial approach to mental disability (cf. Golse, 2013).

Mannoni, in pointing out the psychotic dimension of the family unconscious, simply wished to claim that such an organic causality does not preclude a psychotherapy based on psychoanalytic principles—and that ignoring the unconscious processes emerging in a family upon encountering a child who resembles them so little will prevent his phallicization and thereby prevent the establishment of the cut.

### The lack of cut: the retarded child, caught up in the maternal fantasy

Mannoni starts from the clinical observation according to which the psychologically handicapped child generally encounters a family configuration laden with a “psychotic dimension” (Lacan, 1998a, p. 238), i.e., lacking a Name-of-the-Father: “the handicapped child is rarely welcomed in a genuinely triangular situation” (Mannoni, 1981, p. 32)—that is, acknowledged by the mother as a representative of the father. Therefore, “the lack of a paternal signifier reduces the child to the status of object, without any hope of becoming a subject” (Mannoni, 1981, p. 52): if the mother does not view her child as representing his father (lack of Oedipal S_2_), he becomes a de-phallicized object *a*, not separated by the cut from the maternal Other's body.

The situation is partly related to the effect of the parents' encounter with the psychical handicap of the child. Of course, the extent of the parents' Oedipal organization—and specifically the mother's—plays an important role in the development of the handicapped child; but the effect of his handicap (as early as in ultrasound scans, etc.—cf. Potier, 2009) considerably weakens this organization, and increases the risk of de-phallicization. (The exponential rise of genetic and genomic sequencing nowadays makes this type of clinical configurations an everyday situation: the effects of de-phallicization need to be prevented, in particular, during the announcement consultation consecutive to genetic testing—cf. Potier et al., 2016). In other words, according to Mannoni, an important parental de-phallicization of the child, consecutive to the lack of cut, often occurs even when parents have a neurotic structure (cf. Vanier, 2012, p. 42–43). In these cases, the impact of the handicap on the parental bond is so deep that the mother doesn't relate the child to her desire for the father. It is this de-phallicization dynamics which Lacan aims to single out by using the expression “psychotic dimension” (Lacan, 1998a, p. 238): it does not refer to the specific psychical structure of the mother (or the parents), but to the type of de-phallicized configuration which frequently emerges in the context of a lack of cut characteristic of psychical handicap.

Raymonde, 14 years old, meets Mannoni because of a profound mental retardation and lack of motor coordination, with a “seemingly undeniable organic basis” (Mannoni, 1981, p. 50 sq., as well as all quotes in this paragraph). She rapidly shows a total lack of active resistance or aggressive behavior: everyone is “nice.” All other siblings have a significant academic delay in the absence of mental retardation—and the father is an academic. Her mother is extremely rigid and anxious: she cannot stand the children's liveliness, which frightens her; all of them have personality disorders. Raymonde's mental retardation is a defense against her mother's rigid phobia: “she responds to her mother's demand to not cause any trouble by acting like a nice frightened girl, willing to be forgotten.” The mother explains her severity in potty training by saying “I don't like it when I smell bad”—anamnestic discussions clearly showed that, during pregnancy, she felt as though Raymonde “was part of her own body,” just like “one of her own organs.” This lack of the function of the cut comes from the situation surrounding her pregnancy: the father threatened to leave her in case the child was nonviable: this anticipated lack of acknowledgment from the father, inducing a massive de-phallicization, was assorted (for both parents) with a family structure where each parent's mother sought to exclude their child's partner. Therefore, their own psychical triangulations were already weakened: the father let the mother leave him aside, while she as well had little psychical space for him. This family constellation thus prevented Raymonde from accessing any kind of triangulation. Psychotherapy gradually led to unearth an important persecutory dimension (with spirits invading her body), echoing her mother's hypochondria and her experiences of bodily invasion. Working through these fantasies helped her regain a grip on her mental functioning and her body, which in turn led her to social and professional insertion (she became a gardener working with children). On the other hand, the gradual autonomization enabled by this psychoanalytic process, led Raymonde's mother to a massive disorganization and delusional confusion (Mannoni, 1981) because of her inability elaborate her object *a*'s absence.

In these cases, the desire upon which the child depends is strictly the mother's, “in its most obscure term” (Lacan, 1998a, p. 237): the child is led to adopt the position conferred to him by the maternal fantasy, whatever the latter's precise nature—while he can wonder what he represents as an object *a* for the Other, he does experience the Other's desire as de-phallicized. This is what Mannoni refer to when she writes that in these cases, “as soon as it was conceived, the subject already plays a very specific role in the mother's fantasy; his fate is already sealed; he will be this desireless object whose sole function will be to fill in the maternal emptiness” (Mannoni, 1981, p. 84). This sentence plays on the word “conception”: when the child was physically conceived, he didn't have this status (his handicap was unknown to the parents); but later parental unconscious formations show that, after discovery of the handicap, his conception was fantasmatically re-written into a completely de-phallicized narrative, qualifying him as a non-phallicized object *a* excluded from the couple's desire. “Unbeknownst to him, the child is so to speak ‘caught up’ in the mother's desire. (…) The child's illness will conceal the mother's” (Mannoni, 1981, p. 87). Being caught up means that it is the mother's difficulty to consider separation (this is the “illness”) which often leads her to present her child's illness as the motive of an apparently legitimate care, thereby putting him in the position of an extension of her own body and thus precluding the cut from taking place.

Hereafter, this maternal “illness” (difficulty to consider separation) will lead her to equate separation with losing one's own being: the function of mental retardation “is to hide not only [the child's] lack of being, but what is felt as the mother's lack of being” (Mannoni, 1981, p. 170). A mother tells Mannoni: “since my child left, I feel an emptiness in myself, I don't know what to do with myself, I'm completely at a loss” (Mannoni, 1981, p. 101). For the mother, opening this closed mother-child circuit means self-annihilation: “any claim to autonomy on the part of the child is immediately experienced by the mother as the disappearance of this necessary support of her fantasy” (Mannoni, 1981, p. 86). Correspondingly, the unconscious of the retarded child echoes the mother's emptiness anxiety: “Mother's existence depends on me alone” (Mannoni, 1981, p. 105). This reminds us of the disorganizing effects of Raymonde's autonomization on her mother.

The fantasy refers to the coordinates of the relation to the object, and the object refers to a part of the Other's body, of which the subject must separate himself in order to come to existence. Therefore the term “emptiness” used by this mother can help specify the maternal fantasy wherein the retarded child is caught up: it is an archaic, cannibalistic oral fantasy, where the child-object is being devoured by the mother. Since the child is in a position of non-phallicized object, and is thus not protected from this maternal fantasy by a paternal cut, he experiences this fantasy directly in his body: he is afraid that the Other will snatch and devour him.

“Retarded and psychotic children respond to the threat of the Other with their body. Their body is directly subjected to panic: they lack the symbolic dimension which would help them situate themselves with respect to the Other's desire without risking to be snatched by him” (Mannoni, 1981, p. 198).

The cannibalistic fantasy of devouration had initially been described by Karl Abraham (1916) in the context of the cure of an adult patient; he mostly insisted on the fact that it expresses the infant's oral erotic drive. In the passage above, Mannoni—after Lacan (and Melanie Klein, who was partly trained by Abraham)—insists on the role of the Other in the structure of the fantasy, for two reasons. Firstly, oral drive is, to a large extent, experienced as coming from the Other because of the projective nature of archaic fantasies. And second, the presence of an infant does trigger cannibalistic fantasies in the Other: to some extent, a phallicized infant is protected from the excessive staging or transmission of such fantasies by the presence of the paternal metaphor in the mother's psyche—but in a de-phallicized context such as that of mental retardation, where the infant is experienced as part of her body, he is much less protected from them.

Mannoni points out, on the basis of such clinical material, that this fantasy is prevalent in these children because of the lack of what Lacan conceptualized as paternal cut. Our hypothesis is that, in thus spelling out that this fantasy is at work in the unconscious of mentally retarded children, Mannoni paved the way for Lacan's characterization of infantile symptoms in the late 1960s.

### The first “note on the child” (1986): equating lack of cut with alienation to maternal fantasy

In 1964, in *Seminar XI*, Lacan gives, as the first example of lack of cut, the “psychotic dimension” pointed out by Mannoni in the unconscious of the retarded child caught up in the maternal fantasy. In 1969, when writing the two “Notes on the Child” (given to Jenny Aubry and published for the first time in Aubry, 1983), Lacan draws on Mannoni to generalize her results. From now on, he will describe the effect of a lack of cut in the maternal unconscious (the “psychotic dimension”) with the terms Mannoni used to spell out the family dynamics at work in the background of mental retardation. From now on, the child as object of the maternal fantasy will refer to a type of child symptom, of which mental retardation becomes a specific case.

In the first “Note on the child,” Lacan distinguishes two types of child symptoms, in the psychoanalytic sense of the term—that is, two types of difficulties the child encounters in order to become a subject. In the first case, the symptom can represent the truth of the parental couple, i.e., the encounter of their two Oedipus complexes. Drawing on the unconscious of both parents, it is the most complex case; but it is more open to psychoanalytical work as it relies on a cut—the Name-of-the-Father is present in the mother's unconscious, which thus phallicizes the child. A typical example is Hans' horse-phobia, analyzed by Freud and later by Lacan (1998b): through this phobia, he was working through and gradually integrating the difference of sexes.

In the second case, this symptom is not mediated by the paternal function, i.e., by the Name-of-the-Father: just as with Raymonde's defensive use of her mental retardation, the symptom “stems from the subjectivity of the mother” (Lacan, 1986, p. 13–14), in which case the child is concerned “directly as the correlative of a fantasy” (*id*.). The therapeutic goal then becomes to help the child separate from this dual relationship with the body of the maternal Other [Nowadays, we would most likely use Lacan's indications of two types of symptoms as a spectrum, with being caught up in the maternal fantasy on one end, and representing the truth of the parental couple on the other; or alternatively, as two interacting axes which should both be taken into account. Symptoms such as mental retardation should actually be dealt with drawing on both axes; clinical work with children with milder forms of mental retardation in the context of microdeletion 22q11.2 has made one of us (OP) very sensitive to the de-phallicisation caused by such symptoms. That is, the parental couple, and the subsequent phallicization of the child, can be partly put aside by the mother out of frustration at not having given an ideal phallicized child to her Oedipal father; which can profoundly impact the child's symptoms, in reaction to this disavowal of the importance of the couple].

Typical of this second case are psychosomatic disorders and psychical configurations such as mental retardation—which, for Mannoni, is essentially identical to child psychosis in its structure. Psychosomatic disorders share with mental retardation (and, more generally, psychosis) a lack of paternal metaphor, i.e., both belong to the “psychotic dimension” entailed in being the direct correlative of a maternal fantasy. This doesn't mean that mental retardation is a psychosomatic disorder, but that initially and prior to psychoanalytic work, psychosomatic symptoms are devoid of symbolic meaning: they are characterized by their lack of subjective signification and cannot be related metaphorically to the Oedipal narrative (Lacan, 1998a); as such, they are distinct from hysteric symptoms where bodily affections have an Oedipal signification.

One cannot but be struck to find in Lacan's words (“directly as the correlative of a fantasy”) the exact characterization of the child's being the object of the maternal fantasy because of a lack of cut which Mannoni, partly nourished by her interactions with Lacan, had found in mental retardation when she wrote “the lack of a paternal signifier reduces the child to the status of an object, without any hope of becoming a subject” (Mannoni, 1981, p. 58).

Thus generalizing Mannoni's formulation then allows Lacan to spell out what is entailed in becoming the object of the sole maternal fantasy, and more specifically its de-phallicized object *a*: insofar as the child isn't separated from the mother by the Name-of-the-Father, he “realizes the presence of what Jacques Lacan designates as the object *a* in the fantasy” (Lacan, 1986, p. 13–14)—that is, he appears to the mother as the surrogate to anything she could lack, i.e., she could have lost from the body of her own maternal Other. From this perspective, the mother's subjective structure, determined by a specific “mode of lack” (neurotic, perverse or psychotic), is secondary—the child will nonetheless be put in the position to “saturate” it (Lacan, 1986, p. 13–14), and thereby to conceal from her the truth of her own symptom. In this conception, Lacan takes into account the family dynamics pointed by Mannoni in the case of mental retardation: the child's symptom is the screen of the maternal symptom. Later, during the *RSI* seminar (1974-1975, unpublished), Lacan will refer to the mother's relation to the child saturating her lack with the expression “non-phallic jouissance.”

How can these indications, born from the exchanges between Lacan and Mannoni, be put to work in order to make up for a lack of introduction to the cut by the maternal Other through the paternal metaphor—whether it has led to structures lacking this metaphor (schizophrenic, autistic, etc.) or to severely neurotic ones which somehow made up for this lack? And additionally, what would be a type of cut different from castration, which could be aimed at by psychoanalytic work with non-neurotic patients?

The first theoretico-clinical question was at the core of Mannoni's revolutionary institutional project: in the late 1960s, she founded Bonneuil's Experimental School. Embodying the theoretico-clinical organization of the splintered institution, this School also gave clinical elements toward answering the second question, by helping those of its non-neurotic hosts develop specific types of cuts differing from castration.

## Bonneuil's splintered institution: assuming the cut on the side of the other

### The splintered institution: assuming the cut by acknowledging projects as S_2_

When the mothering Other's unconscious hasn't exposed the child to the Name-of-the-Father, but has instead subjected him to her sole fantasy, the Other's comings and goings in the *fort-da* cannot be understood by the child as manifesting a desire for someone else. Confronted with a maternal unconscious seeking to saturate her lack by reducing the child to the status of non-phallic object *a*, the child experiences separation as potential destruction of the Other – by fragmenting, exploding or emptying her body (cf. the ≪ emptiness ≫ feeling mentioned to Mannoni). Correspondingly, the infant identifies with this experience and fears his own explosion once separated from the other.

Clinical work with children not previously introduced to the cut through the paternal metaphor will thus have to make them feel that it is actually possible to separate from the maternal Other without destroying her—and thus, without risking their own life, for they are not part of her body. Insofar as their type of transference is—at least initially—dual (staging a face-to-face struggle with an almighty Other), Mannoni suggested an institutional therapeutic setting in order to provide, in material reality, a constant and permanent containment for their anxieties. But in order to prevent the institution (with its constant presence) from becoming the transferential replica of an almighty Other about to devour her child-object in a dual relationship, and instead to enable children to access the cut, she put forward a new type of therapeutic institution: the splintered institution, which assumes the cut and thereby makes separation possible on the part of the child.

“An institution is like a person feeding off of those who depend on her. It practically assumes an almighty position: it behaves likes the mother of a psychotic child, from whom the subject cannot separate without risking to explode. A different institution would, much like a scale, assume the cut and thereby make it possible for the subject to situate herself through his own speech—and to thus separate himself, cut himself off of the institution (…). The cut becomes possible, exactly as with a mother and her child. The cut is a symbolic phenomenon, which allows the subject to emerge and be acknowledged as such by someone else” (Mannoni, 1976, p. 53).

To prevent the establishment of a psychotic transference with respect to the institution—which would equate separation with mutual destruction—due to a lack of cut in the child's psyche, the strategy would be to take up any wish of the children for a project outside the institution (trip, activity, etc.), and consider it as an attempt at establishing a cut. “Assuming the cut,” as Mannoni put it, means that any expression of such a project-wish is to be “acknowledged” by the institution staff as a potential S_2_, i.e., as a symbolic representation of the child as bearing a different identity (taking-part-in-such-and-such-project) and thereby as implementing the function of the cut in their psyche, thus allowing them to withstand separation from the Other's body. By offering them the figure of a non-devouring maternal Other, who can bear separation by deliberately cutting herself from them in response to their project-wish, the institution re-enacts the *fort-da* with a different outcome: physical separation in the course of the project doesn't amount to mutual destruction, as it is rather the implementation of the cut—i.e., the result of the symbolic acknowledgment by the Other of a new identity for the child, different from that of de-phallicized object *a*. Taking up the child's wish enables the institution to present him with a different desire of the Other with respect to him, and to represent himself as absent from him.

Mannoni proposes to call such an institution a “splintered institution” (“institution éclatée,” in French—cf. e.g. Mannoni, 1973, p. 77): “splinter” explicitly refers to the psychotic fantasy (characteristic of what Lacan called the “psychotic dimension”) which identifies loss of a bodily part with explosion and annihilation. By coining this expression, Mannoni wanted to stress that it should be the task of the institution itself to take up and overcome the Other's annihilation anxiety associated with loss of a bodily part, which children want to prevent by remaining attached to their status of his de-phallicized objects *a*, by responding positively to their expressed project-wishes (symbolic acknowledgment of the cut) and showing them that it can withstand their outcome (physical separation).

Mannoni's project of a splintered institution, which would “assume the cut,” quickly became the motto of the Experimental School situated in Bonneuil-sur-Marne (just outside of Paris), founded in 1969 by Maud Mannoni and Robert Lefort, both students of Lacan's, with a couple of educators, Rose-Marie and Yves Guérin. The School is a place where children and adolescents live and take classes adjusted to their capacities; it also is a night shelter. Today, it is a daycare hospital and therapeutic night foster home. Schooling is in small groups, and goes along with creative activities supervised by educators; children do their part in the maintenance and daily chores.

The School rapidly became what Mannoni theorized as the splintered institution by considering the children's speech as wishes: far from being a technique, its organization revolves around the fundamental psychoanalytic tenet to start from what the subject says—that is, to always consider the person as a subject, and what they say as a manifestation of their subjectivity (see the section Conclusion below). The goal being to help children bear separation, it is a negative therapy of sorts, the goal of which is to constrain as little as possible. For example, in the early 1970s, an educator suggested to one of the adolescents, upon seeing his gift in bike repair, that he create a repair studio within the institution. The response was that he'd much rather work in a real bike repair shop (potential S_2_), outside the institution. The adolescent's refusal, embedded within his transference on the educator, made it possible for him, first, to enact the rejection of an unconscious almighty Other. By taking up the educator's acknowledgment of his skills, he could then voice a true potential S_2_, to which the institution had to respond by allowing him to exist under this new identity; this gave a new meaning to this presence in the institution, as a moment embedded within his particular project. By acknowledging this new representation of himself, the institution helped him implement the psychical function of the cut and thus envision himself as physically separated from the Other: the *fort-da* could be undergone without anxiety of mutual annihilation.

“The *Fort-Da* play, this oscillation between a here and a there, is introduced in Bonneuil every time a child's stay involves an alternation with moments spent somewhere else” (Mannoni, 1976, p. 73).

In this light, projects such as going abroad are particularly interesting experiences: the immersion in a foreign language deepens the inscription in a new potential S_2_, and allows for another representation of the desire of the Other.

With respect to families, the institution's whole clinical challenge then became to prevent the closure dynamics which can appear in response to the implementation of the cut in the child's psyche, because of the repetition compulsion which prevents them from letting the child escape his alienation to the Other's almighty fantasy and thus access a potential new S_2_. Mannoni has the following exchange with the mother of a patient who cannot but try and thwart Bonneuil's dynamics:

“Julien's mother [JM]: Brittany does wonders for Julien.

Maud Mannoni [MM]: Yes, but it is of crucial importance that you do not settle here, or else we'll have to find him another place to escape the family…

JM: You think so ? I wanted to move here with his twin brother.

MM: We've been lucky enough to find a place Julien likes, where he can enact his rejection of both his family and Bonneuil. It is his own place, where his mother and brother are absent. This is of the utmost symbolic importance; you're about to rob him of this.

JM: The host family had even agreed to accept his twin brother…

MM: You are inducing a potential failure, here. You know too well that, when he's with you, the situation escalates very quickly.

JM: After fifteen minutes, he starts insulting me, while he's normal and composed with everyone else.

MM: And yet, it is this very hell that you seek to re-create.

JM: No, but why would he be normal with others and not with me?

MM: Why hunt him down to the place where he feels at peace, without his family?

JM: It's not on purpose that I knock everything down. I just can't help it” (Mannoni, 1976, p. 225).

Mannoni firmly tries to hold the symbolic function of the cut (by stressing that the project is Julien's own), in order to protect both mother and child from the sado-masochistic dual relationship which re-emerges when they are together. Mannoni expands on this symbolic function by stressing that the movement is to be understood as a variation of frames (a direct reference to the transferential setting), i.e., as a symbolic oscillation.

“To offer another place is to build an alternative to the logics of rejection, to escape the deadlock of the inside-outside opposition with respect to the family or the institution, by favoring a movement between different places. By playing with different frames, we re-introduce a movement within the inside-outside opposition, and the subject can gradually reflect upon what he wants to become” (Mannoni, 1986, p. 106).

Distanced from the S_1_ of his illness which feeds the fantasy of a devouring Other on which the child depends, the S_2_ acknowledged by the institution represents a discourse which can make up for the initial lack of paternal metaphor and implement a cut, thus leading the child or adolescent to withstand physical separation.

### Varieties of cut

The goal of the splintered institution is thus to make up for the initial lack of paternal metaphor, and subsequently of castration—the type of cut consecutive to this metaphor. But this doesn't mean that castration is the only type of cut that this type of institution sets out to enable by acknowledging children's projects. A potential S_2_, being implemented as a function of the cut through institutional support will not necessarily amount to an Oedipal narrative; to that effect, the subject would need to be in the coordinates of a mostly neurotic structuration. This narrative is but one type of S_2_ enabling the cut by representing the subject *qua* the object that the Other lacks, i.e., is separated from—and therefore desires. It is one way to symbolically account for the subject's physical separation from the Other—by drawing on triangulation, i.e., the presence of someone else as object of desire. As mentioned earlier, castration accounts for this specific type of cut because it binds the subject to the desire of the couple constituted by the Other and the bearer of the Name-of-the-Father.

In the splintered institution, the type of cut will depend on the psychical structure of each subject: an attempt as symbolically representing separation can use other means, such as delusion—a highly symbolic production. One need only think of Schreber's delusional narrative (Freud, 1958), structured around the fantasy of becoming God's wife (S_2_), the object that the He needs to become complete. The cut from the Other that Schreber gradually elaborates is not symbolic castration, but it is nonetheless an attempt at building an alternative to the fantasy of being annihilated by an almighty Other, through becoming the phallicized object that He needs (and thus cannot annihilate).

To give an example of alternate type of cut encountered at Bonneuil (collected and synthesized by AV, a close collaborator of Mannoni's), work with autistic children revolved around helping them realize that their project, acknowledged as S_2_ by the team, involved physical separation—which, in autism, amounts to death by annihilation, and is thus to be avoided at all costs. In other words, the focus of the function of the cut to be enabled by the institution was the acknowledgment of physical separation as such; it is the earliest type of cut being staged in the *fort-da*, prior to any narrative accounting for it. Taking up their wishes to go outside for various projects was successful in psychically implementing the cut when, upon coming back to Bonneuil from the countryside, they suddenly cried upon realizing that they had left behind their “family.” Representing themselves as separated from them, that is as having been lost by them, could be used as a shifting point to be drawn upon in the gradual working through of physical separation at stake in the *fort-da*—which is quite different from the neurotic cut *qua* symbolic castration, mostly revolving around elaborating Oedipal issues.

## Conclusion

We tried to show the fruitfulness of Lacan's concept of cut by addressing it from the perspective of the psychical configurations where it hasn't been implemented (with the child being put in the position of a de-phallicized object *a*). Maud Mannoni has explored these configurations extensively: therefore our goal was, in part 2 *supra*, to show how she has characterized some of them, which then helped Lacan define them in a systematic fashion. Finally, we wanted to lay out how Mannoni delineated a theoretico-clinical setting aimed at making up for a lack of cut, as defined by Lacan, and enabling it when subjectively possible (part 3 *supra*).

Mannoni's original use of this concept of cut in the splintered institution has been extended in an original fashion by one of us (AV) in the context of a hospital work with psychotic mothers and their infant; this led him to the idea of a “supposition of subject” (Vanier, 1989). The gist of it is that, in the presence of a psychotic type of mothering, the neonate's psychical development (and indeed, his effective survival) will compensate the mother's tendency to consider him as part of her body, by assuming the function of the cut and acknowledging him as a subject from the start—that is, as someone with a potential to be embedded in various S_2_.

## Author contributions

LR has provided much of the material concerning Mannoni, and has put forward the importance of movement; she has contributed to the structure of the paper. OP has provided much of the material concerning Lacan. He has organized the global structure of the paper, and has written the detailed version of the paper. AV has contributed to writing the paper (material concerning Lacan and Mannoni, structure of the paper, references). All authors read and approved the final manuscript.

### Conflict of interest statement

The authors declare that the research was conducted in the absence of any commercial or financial relationships that could be construed as a potential conflict of interest.
